# Cross-sectional associations and incremental discrimination of inflammatory and lipid-derived indices for prevalent coronary heart disease in type 2 diabetes

**DOI:** 10.3389/fendo.2026.1961170

**Published:** 2026-09-24

**Authors:** Changlin Xie, Lingjiao Meng, Yifan Wang, Yilin Wang, Haijiao Shi

**Affiliations:** 1 Liaoning University of Traditional Chinese Medicine, Shenyang, Liaoning, China; 2 The Affiliated Hospital of Liaoning University of Traditional Chinese Medicine, Shenyang, Liaoning, China

**Keywords:** coronary heart disease, cross-sectional association, inflammatory indices, internal validation, systemic immune-inflammation index, type 2 diabetes mellitus

## Abstract

**Background:**

Routine blood-count and lipid-derived indices may be associated with coronary heart disease (CHD) in type 2 diabetes mellitus (T2DM), but comparative evidence within one cohort is limited.

**Methods:**

This cross-sectional study included 3,964 adults with T2DM. Six inflammatory and three lipid-derived indices were examined using quartile-based and standardized continuous logistic regression. Domain-specific covariate sets avoided adjustment for constituent lipids. Dose–response, subgroup, and temporal sensitivity analyses were performed. Incremental discrimination was assessed against a common clinical base model with bootstrap optimism correction. Calibration and decision curves were based on cross-validated estimated probabilities; high-sensitivity C-reactive protein was a benchmark.

**Results:**

CHD was present in 1,350 participants (34.1%). Median SII was 649 × 10^9^/L in participants with CHD and 522 × 10^9^/L in those without CHD (*P* < 0.001). The adjusted OR for SII was 2.37 (95% CI, 1.91–2.93) for Q4 versus Q1 and 1.46 (95% CI, 1.36–1.58) per 1-SD increase. The association was nonlinear (*P* for nonlinearity < 0.001), with no interaction by sex, age, BMI, or hypertension. The optimism-corrected AUC increased from 0.729 to 0.744 after adding SII (ΔAUC = 0.015, *P* < 0.001); corrected AUCs were 0.744 for PIV and 0.745 for SIRI. Paired comparisons showed no statistically significant difference between the SII-augmented model and either the PIV-augmented model (P = 0.964) or the SIRI-augmented model (P = 0.717). The SII model showed a cross-validated AUC of 0.742, Brier score of 0.188, ICI of 0.017, and calibration slope of 0.963.

**Conclusion:**

Several indices were associated with prevalent CHD, without consistent evidence that SII was superior.

## 1 Introduction

Recent Global Burden of Disease estimates indicate that approximately 254 million people were living with ischemic heart disease globally in 2021, underscoring its substantial health and socioeconomic burden (1, 2). Longitudinal evidence suggests that sustained adiposity exposure is associated with mortality among adults with T2DM, with a sustained BMI ≥27 kg/m² associated with increased mortality and longer maintenance of BMI within 18.5–26.9 kg/m² associated with improved survival (3).

Chronic low-grade inflammation contributes to multiple stages of atherosclerosis, including endothelial dysfunction, leukocyte recruitment, foam-cell formation, plaque progression, and plaque destabilization (4). Composite inflammatory indices derived from routine complete blood counts combine information from neutrophils, lymphocytes, monocytes, and platelets and may capture different aspects of systemic inflammatory and immune activity (5). The systemic immune-inflammation index (SII), initially developed as a prognostic marker in oncology, has subsequently been associated with cardiovascular disease and adverse cardiovascular outcomes (6). Other CBC-derived indices—including the neutrophil-to-lymphocyte ratio (NLR), platelet-to-lymphocyte ratio (PLR), lymphocyte-to-monocyte ratio (LMR), systemic inflammation response index (SIRI), and pan-immune-inflammation value (PIV)—have also been associated with coronary atherosclerosis or cardiovascular outcomes in different populations (7–9). However, these indices share several hematologic components and are therefore likely to provide substantially overlapping information.

Lipid-derived indices may capture complementary aspects of atherogenic dyslipidemia and insulin resistance. The triglyceride-to-high-density lipoprotein cholesterol ratio (TG/HDL-C), non-high-density lipoprotein cholesterol (non-HDL-C), and atherogenic index of plasma (AIP) have each been associated with coronary atherosclerosis or cardiovascular outcomes. In particular, AIP has been associated with angiographically defined coronary artery disease among patients with T2DM (10). Nevertheless, AIP is a logarithmic transformation of TG/HDL-C, and all three lipid-derived indices are mathematically dependent on conventional lipid measurements. Their associations may therefore be distorted if regression models simultaneously adjust for their constituent lipids.

Although these inflammatory and lipid-derived indices have frequently been examined individually, direct comparisons within the same T2DM population remain limited. Moreover, the magnitude of a cross-sectional association is not equivalent to incremental model discrimination, and comparisons based only on point estimates may be misleading when the indices are strongly correlated. Accordingly, this study aimed to compare nine routinely available indices—SII, PIV, SIRI, NLR, PLR, LMR, AIP, TG/HDL-C, and non-HDL-C—in relation to the odds of prevalent CHD among adults with T2DM. We examined quartile-based and standardized continuous associations, dose–response patterns, and correlations among the indices, and evaluated their incremental discrimination when added individually to a common clinical base model, with high-sensitivity C-reactive protein included as a benchmark comparator and internal validation used to account for optimism.

## 2 Materials and methods

### 2.1 Study design and population

This single-center, cross-sectional study used electronic medical record data from the Affiliated Hospital of Liaoning University of Traditional Chinese Medicine, Liaoning, China, between January 2023 and December 2024. Consecutive inpatient and outpatient encounters involving adults aged ≥18 years with type 2 diabetes mellitus (T2DM) were screened. T2DM was identified according to a documented physician diagnosis or the diagnostic criteria of the American Diabetes Association applied during routine clinical care.

Patients were eligible when they had a fasting complete blood count, conventional lipid profile, glycated hemoglobin (HbA1c), serum creatinine, high-sensitivity C-reactive protein (hs-CRP), sufficient clinical and medication information, and records permitting ascertainment of coronary heart disease (CHD) status. Patients were excluded for type 1 diabetes, gestational diabetes, or another specific type of diabetes; severe renal impairment, defined as end-stage kidney disease, current dialysis, estimated glomerular filtration rate (eGFR) <30 mL/min/1.73 m², or physician-documented severe renal failure; decompensated cirrhosis, hepatic failure, or other physician-documented severe hepatic impairment; an autoimmune or hematologic disorder likely to affect peripheral blood-cell counts; active malignancy; active acute infection at the index encounter; or unavailable variables required to calculate the study indices or fit the multivariable models.

Among patients meeting more than one exclusion criterion, one primary reason was assigned in the following hierarchical order: non-T2DM diabetes type, severe renal or hepatic impairment, autoimmune or hematologic disorder, active malignancy, acute infection, and incomplete clinical or laboratory data. The source population comprised 4,720 patients; 756 were excluded and 3,964 were retained in the complete-case analytical cohort, as shown in **Figure 1**. The study was approved by the Institutional Review Board of the Affiliated Hospital of Liaoning University of Traditional Chinese Medicine (Approval No. Y2025305CS(KT)-305-01). The requirement for informed consent was waived because only de-identified routinely collected data were analyzed.

**Figure 1:**
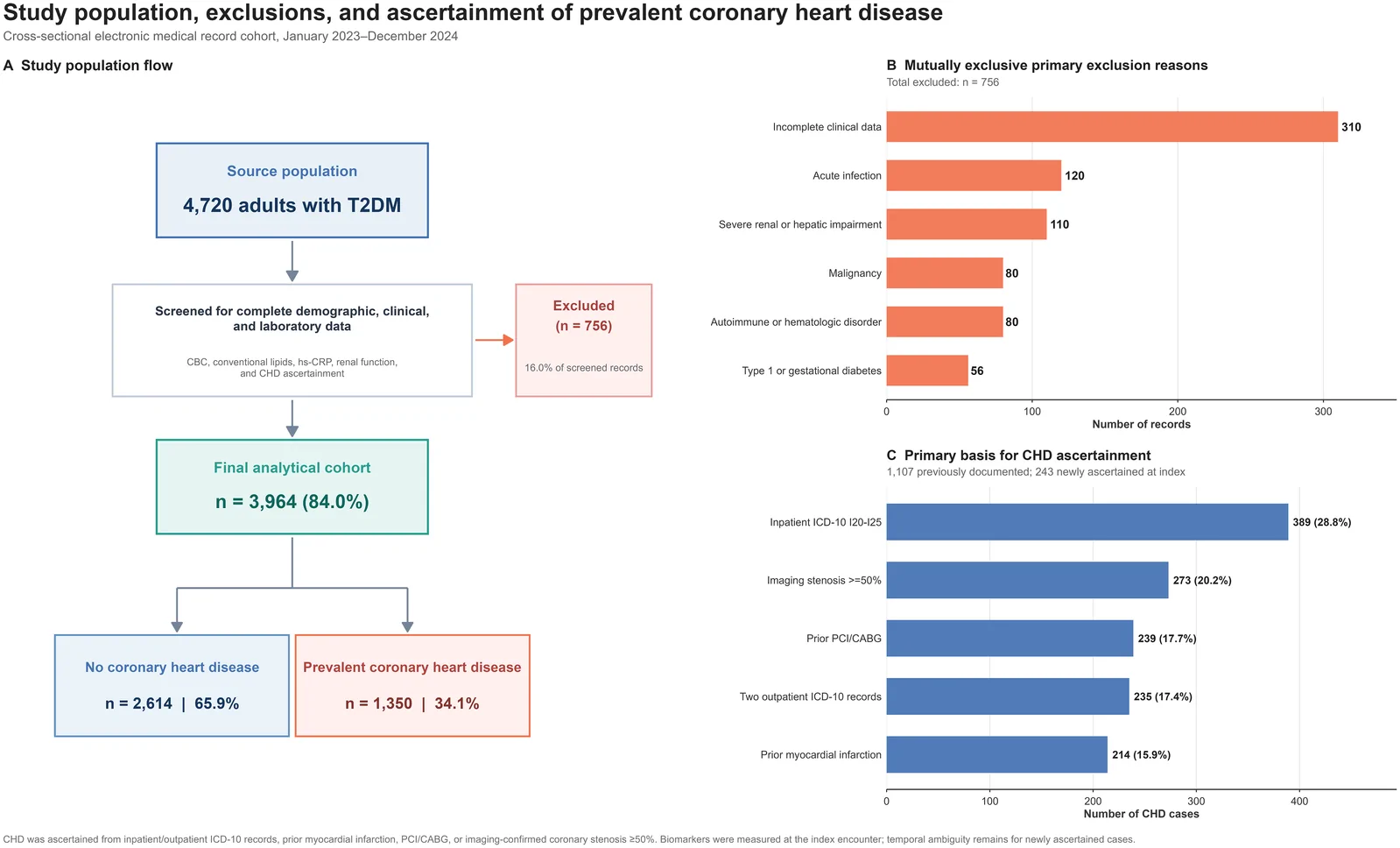
Study population, exclusions, and ascertainment of prevalent coronary heart disease. (**A**) Selection of the final analytical cohort from 4,720 adults with T2DM. **(B)** Mutually exclusive primary exclusion reasons. **(C)** Mutually exclusive primary basis for CHD ascertainment among 1,350 cases. Biomarkers were measured at the index encounter; temporal ambiguity remains for newly ascertained cases. CABG, coronary artery bypass grafting; CHD, coronary heart disease; PCI, percutaneous coronary intervention; T2DM, type 2 diabetes mellitus.

### 2.2 Index encounter and measurement timing

The index encounter was defined as the earliest chronologically eligible inpatient or outpatient encounter during the study period at which the required fasting laboratory measurements and clinical information were simultaneously available. Only one index record was retained for each patient. Complete blood counts, lipid measurements, HbA1c, serum creatinine, and hs-CRP were obtained from samples associated with the same index laboratory date. Recorded fasting durations ranged from 8 to 14 hours.

For patients with previously documented CHD, the first recorded CHD date preceded the index laboratory date. CHD first confirmed during the index encounter, including confirmation by coronary angiography or coronary computed tomography angiography performed within seven days after the index laboratory assessment, was classified as newly ascertained CHD. Consequently, the biomarker measurements were treated as cross-sectional exposure measurements associated with prevalent CHD rather than measurements obtained before disease onset.

### 2.3 Data collection and clinical definitions

Demographic and clinical variables included age, sex, body mass index (BMI), diabetes duration, systolic and diastolic blood pressure, hypertension, dyslipidemia, smoking status, current alcohol consumption, and encounter type. Smoking was classified as never, former, or current smoking. Current drinking was defined as alcohol consumption at least once per week. Medication use was determined from medications documented as active at the index encounter and included glucose-lowering, antihypertensive, lipid-lowering and antiplatelet therapies, insulin, metformin, sodium-glucose cotransporter-2 inhibitors, glucagon-like peptide-1 receptor agonists, and systemic glucocorticoids.

Hypertension was defined as systolic blood pressure ≥140 mmHg, diastolic blood pressure ≥90 mmHg, or current antihypertensive treatment. Dyslipidemia was defined as total cholesterol ≥5.2 mmol/L, triglycerides ≥1.7 mmol/L, LDL cholesterol (LDL-C) ≥3.4 mmol/L, HDL cholesterol (HDL-C) <1.0 mmol/L in men or <1.3 mmol/L in women, or current lipid-lowering treatment. eGFR was calculated from serum creatinine using the 2021 CKD-EPI creatinine equation without a race coefficient.

Laboratory measurements included fasting glucose, HbA1c, serum creatinine, alanine aminotransferase, aspartate aminotransferase, total cholesterol, triglycerides, HDL-C, LDL-C, hs-CRP, total white blood cell count, neutrophil count, lymphocyte count, monocyte count, eosinophil count, basophil count, and platelet count. Measurements were performed by the hospital clinical laboratory under routine standardized specimen-processing and internal quality-control procedures. Analyzer-specific information was unavailable in the de-identified analytical dataset and was therefore not used as an analytical criterion.

### 2.4 Calculation of inflammatory and lipid-derived indices

Six inflammatory indices were calculated from absolute peripheral blood-cell counts. The systemic immune-inflammation index (SII) was calculated as platelet count × neutrophil count/lymphocyte count and expressed as ×10^9^/L. The neutrophil-to-lymphocyte ratio (NLR) was calculated as neutrophil count/lymphocyte count; the platelet-to-lymphocyte ratio (PLR) as platelet count/lymphocyte count; and the lymphocyte-to-monocyte ratio (LMR) as lymphocyte count/monocyte count. The systemic inflammation response index (SIRI) was calculated as neutrophil count × monocyte count/lymphocyte count, and the pan-immune-inflammation value (PIV) as neutrophil count × platelet count × monocyte count/lymphocyte count.

Three lipid-derived indices were calculated. The triglyceride-to-HDL cholesterol ratio (TG/HDL-C) was defined as triglycerides divided by HDL-C, with both measurements expressed in mmol/L. The atherogenic index of plasma (AIP) was calculated as log_10_(TG/HDL-C). Non-HDL cholesterol (non-HDL-C) was calculated as total cholesterol minus HDL-C and expressed in mmol/L.

Nine indices were therefore evaluated: SII, PIV, SIRI, NLR, PLR, LMR, AIP, TG/HDL-C, and non-HDL-C. Because AIP is exactly a logarithmic transformation of TG/HDL-C, the two indices contain identical ranking information. Both were retained as separately named clinical indices for transparent reporting, but they were not interpreted as independent biomarkers.

### 2.5 Ascertainment of prevalent coronary heart disease

The primary outcome was prevalent CHD at or in relation to the index encounter. CHD was considered present when at least one of the following criteria was satisfied: an inpatient encounter carrying an International Classification of Diseases, Tenth Revision code from I20 to I25; at least two outpatient encounters carrying an I20-I25 code on different dates within a two-year period; documented previous myocardial infarction; previous percutaneous coronary intervention or coronary artery bypass grafting; or coronary stenosis ≥50% documented by invasive coronary angiography or coronary computed tomography angiography.

CHD status was derived from structured diagnostic, procedural, historical, and imaging fields in the electronic medical record. Diagnostic evidence categories were allowed to overlap because individual patients could satisfy more than one criterion. A separate mutually exclusive primary-criterion variable recorded the criterion used to classify the principal basis of each CHD case, while the individual evidence variables were retained for describing the clinical composition of the outcome. No independent external event-adjudication committee was used.

The date of the first qualifying CHD record was compared with the index laboratory date. Patients whose first qualifying record preceded the laboratory date were classified as having previously documented CHD. Patients first meeting a qualifying criterion on the laboratory date or within the subsequent seven days of the same diagnostic episode were classified as having CHD newly ascertained at the index encounter. Acute coronary syndrome at the index encounter was determined from the corresponding structured clinical diagnosis recorded for that encounter.

### 2.6 Missing data and cohort selection

A complete-case analytical approach was used, and no statistical imputation was performed. Patients were excluded for incomplete data when at least one variable required to calculate the inflammatory or lipid-derived indices, determine CHD status, or fit the specified multivariable models was unavailable. All variables included in the primary analyses were complete in the final analytical cohort. CHD-specific variables, including the first documented CHD date and principal ascertainment criterion, were structurally inapplicable to participants without CHD and were not classified as missing data.

Missingness of core variables was quantified in the source population before complete-case selection. To assess the possibility of selection bias, available demographic, clinical, renal, lipid, hs-CRP, and SII measurements were compared between included and excluded records. Primary exclusion reasons were summarized as mutually exclusive categories to prevent double counting.

### 2.7 Descriptive analyses

Continuous variables were summarized as mean ± standard deviation when approximately normally distributed and as median with interquartile range when skewed. Categorical variables were reported as counts and percentages. Between-group comparisons used Welch’s t test for approximately normally distributed continuous variables, the Wilcoxon rank-sum test for skewed continuous variables, and the chi-square or Fisher exact test for categorical variables, as appropriate.

Absolute standardized mean differences were calculated to describe differences in characteristics between patients with and without prevalent CHD and between included and excluded records. For skewed biomarkers, standardized mean differences were calculated on the transformed scale. Standardized mean differences were interpreted as measures of group imbalance rather than hypothesis tests.

### 2.8 Logistic regression analyses

Each index was divided into four empirical, sample-specific quartiles of approximately equal size, with the lowest quartile used as the reference. Quartile membership was assigned from participant ranks to avoid ambiguity caused by values equal to empirical cut points. Because AIP is a monotonic transformation of TG/HDL-C, both indices used the same participant ranking and quartile membership. Linear trends were tested by entering quartile category as an ordinal score from 1 to 4.

Continuous analyses were performed on a common standardized scale. SII, PIV, SIRI, NLR, PLR, LMR, TG/HDL-C, and non-HDL-C were log_2_-transformed and subsequently standardized to a mean of zero and standard deviation of one. AIP, which was already defined on a logarithmic scale, was standardized without an additional log_2_ transformation. Continuous ORs therefore represented the change in the odds of prevalent CHD per 1-SD increase in the transformed index.

Binary logistic regression was used to estimate odds ratios (ORs) and 95% confidence intervals (CIs). Model 1 was unadjusted, and Model 2 was adjusted for age and sex. Model 3 used domain-specific covariate sets. All Model 3 analyses included age, sex, BMI, diabetes duration, hypertension, smoking status, current drinking, HbA1c, fasting glucose, eGFR, and use of glucose-lowering, antihypertensive, and lipid-lowering medications. Models for the six inflammatory indices additionally included triglycerides, HDL-C, and LDL-C. Models for AIP, TG/HDL-C, and non-HDL-C excluded conventional lipid measurements to avoid adjustment for variables that were mathematical components of the exposure. ORs were interpreted as associations with the odds of prevalent CHD and not as risk ratios.

### 2.9 Correlation and dose-response analyses

Dependence among the nine indices and hs-CRP was evaluated using Spearman rank-correlation coefficients. AIP and TG/HDL-C were expected to have a Spearman correlation of 1 because one is a monotonic transformation of the other.

Adjusted dose-response relationships were evaluated for all nine indices using natural cubic spline models with three degrees of freedom and the median index value as the reference. The spline basis was generated using the default quantile-based placement implemented in R. Models used the corresponding domain-specific Model 3 covariates. Evidence of nonlinearity was evaluated using a likelihood-ratio test comparing the spline model with a model containing only the linear exposure term. Curves were restricted to the central 90% of each index distribution to reduce the influence of sparse extreme observations.

### 2.10 Subgroup and sensitivity analyses

Subgroup analyses of SII were performed according to sex, age (<65 or ≥65 years), BMI (<24, 24 to <28, or ≥28 kg/m²), and hypertension status. SII was modeled per 1-SD increase in log_2_-transformed values. The domain-specific Model 3 covariates were retained, except that the corresponding stratification variable was not included when it was constant within a stratum. Multiplicative interaction terms were tested in the full analytical cohort, and global interaction *P* values were obtained using likelihood-ratio tests.

Three sensitivity analyses addressed temporal ambiguity and potential reverse causation. The adjusted continuous SII analysis was repeated after excluding patients with acute coronary syndrome at the index encounter, after excluding all CHD cases newly ascertained at the index encounter, and after restricting CHD cases to those documented at least 365 days before the index laboratory measurement. These analyses used the same covariate set and exposure transformation as the primary continuous SII model.

### 2.11 Internal validation and incremental discrimination

Incremental discrimination was evaluated by adding each of the nine indices separately to a common clinical base model. The base model included age, sex, BMI, diabetes duration, hypertension, smoking status, current drinking, HbA1c, fasting glucose, eGFR, and use of glucose-lowering, antihypertensive, and lipid-lowering medications. Conventional lipid measurements were excluded from the common base model to permit comparable assessment of lipid-derived indices without mathematical coupling. hs-CRP was evaluated separately as a benchmark comparator.

Discrimination was quantified using the area under the receiver-operating-characteristic curve (AUC). Apparent AUC estimates were corrected for optimism using 500 nonparametric participant-level bootstrap samples. In each bootstrap iteration, a sample equal in size to the original cohort was drawn with replacement. Identical resampling indices were used for the clinical base model and all marker-augmented models to maintain paired comparisons. Each model was refitted in the bootstrap sample, and its AUC was calculated both in the bootstrap sample and in the original cohort using predictions from the same bootstrap-fitted model. Model-specific optimism was defined as the AUC in the bootstrap sample minus the corresponding test AUC in the original cohort. The optimism-corrected AUC was calculated by subtracting the mean optimism across the 500 iterations from the apparent AUC in the original cohort.

For each marker-versus-base comparison, the apparent ΔAUC was calculated as the original-sample AUC of the marker-augmented model minus that of the clinical base model. Within each bootstrap iteration, the paired ΔAUC was calculated separately in the bootstrap sample and in the original cohort using the corresponding bootstrap-fitted models. Optimism in ΔAUC was defined as the paired ΔAUC in the bootstrap sample minus the paired test ΔAUC in the original cohort. The optimism-corrected ΔAUC was calculated by subtracting the mean optimism in ΔAUC from the original-sample apparent ΔAUC. Direct comparisons between the SII-augmented model and each comparator model used the same participant-level bootstrap samples and an analogous paired procedure.

Location-shifted 95% confidence intervals were obtained by shifting the 2.5th and 97.5th percentiles of the bootstrap distribution of the apparent paired ΔAUC by the estimated mean optimism. Paired-bootstrap standard errors were calculated as the standard deviations of the corresponding location-shifted bootstrap distributions. Two-sided P values were calculated using a normal approximation based on the optimism-corrected ΔAUC estimate and its paired-bootstrap standard error.

For the SII-augmented model, out-of-sample predicted probabilities were generated using 10-fold cross-validation stratified by CHD status. These predictions were used to calculate the cross-validated AUC, Brier score, integrated calibration index, calibration intercept, and calibration slope. Calibration was assessed across deciles of predicted probability and using a locally weighted regression curve. Decision-curve analysis used the cross-validated probabilities to compare the net benefit of the clinical base model and models augmented with SII, PIV, SIRI, or hs-CRP against treat-all and treat-none strategies over threshold probabilities from 5% to 60%. Continuous NRI and IDI were not used because of their sensitivity to model calibration and potential upward bias.

### 2.12 Statistical software and inference

All analyses were performed using R version 4.6.0 (R Foundation for Statistical Computing, Vienna, Austria). A fixed random seed of 20260828 was used for bootstrap resampling and cross-validation. Statistical tests were two-sided, and nominal *P* values <0.05 were considered statistically significant. No multiplicity adjustment was applied. The nine-index comparison and three-model adjustment sequence were not prospectively registered; analyses involving multiple indices, spline tests, subgroups, sensitivity analyses, and pairwise model comparisons were therefore treated as exploratory, and all reported P values were nominal.

## 3 Results

### 3.1 Cohort selection, missing data, and CHD ascertainment

A total of 4,720 adults with T2DM were screened, of whom 756 were excluded. The mutually exclusive primary reasons for exclusion were incomplete clinical data in 310 patients (41.0% of excluded records), acute infection in 120 (15.9%), severe renal or hepatic impairment in 110 (14.6%), an autoimmune or hematologic disorder in 80 (10.6%), malignancy in 80 (10.6%), and type 1 or gestational diabetes in 56 (7.4%). The final complete-case analytical cohort comprised 3,964 participants, including 2,614 without CHD and 1,350 with prevalent CHD, corresponding to a CHD prevalence of 34.1% (**Supplementary Table S1A**).

Missingness of individual core variables in the source population ranged from 0.0% to 3.0%. Age, sex, hypertension, and smoking status were complete, whereas LDL-C had the highest proportion of missing values (140/4,720, 3.0%), followed by eGFR (135/4,720, 2.9%). Compared with included participants, excluded participants had lower mean eGFR (71.4 ± 31.7 vs. 84.3 ± 18.6 mL/min/1.73 m²; absolute SMD = 0.50) and higher median hs-CRP [3.03 (1.04–8.65) vs. 2.05 (0.88–4.59) mg/L; absolute SMD = 0.46]. The remaining reported demographic, clinical, lipid, and inflammatory characteristics were similar between included and excluded records, with absolute SMDs ≤0.04; median SII was 558 (400–773) and 559 (401–799) ×10^9^/L, respectively (absolute SMD = 0.02) (**Supplementary Tables S1B, C**).

Among the 1,350 CHD cases, the mutually exclusive primary ascertainment criteria were an inpatient ICD-10 I20–I25 record in 389 patients (28.8%), imaging-confirmed coronary stenosis ≥50% in 273 (20.2%), previous PCI or CABG in 239 (17.7%), at least two outpatient ICD-10 records in 235 (17.4%), and previous myocardial infarction in 214 (15.9%). CHD had been documented before the index encounter in 1,107 cases (82.0%), whereas 243 cases (18.0%) were newly ascertained at the index encounter. Because diagnostic evidence could overlap, 52.8% of CHD cases had an inpatient ICD-10 record, 49.3% had imaging-confirmed stenosis, 39.9% had undergone PCI, 36.2% had at least two outpatient diagnostic records, 35.0% had a history of myocardial infarction, and 7.0% had undergone CABG; 135 patients (10.0%) had acute coronary syndrome at the index encounter (**Supplementary Tables S2A–C**).

### 3.2 Baseline characteristics according to prevalent CHD status

Participants with prevalent CHD were older than those without CHD (66.7 ± 10.3 vs. 60.7 ± 10.5 years; absolute SMD = 0.58), and a greater proportion were male (61.1% vs. 48.2%; absolute SMD = 0.26). They also had a higher mean BMI (26.5 ± 3.9 vs. 25.6 ± 3.7 kg/m²), longer diabetes duration (10.9 ± 6.8 vs. 8.3 ± 6.1 years), and higher systolic blood pressure (137.8 ± 15.9 vs. 131.8 ± 15.9 mmHg). Hypertension (68.9% vs. 46.3%; absolute SMD = 0.47), dyslipidemia (55.9% vs. 42.8%), and current smoking (29.3% vs. 23.5%) were more frequent among participants with CHD, whereas current drinking was comparable between groups (21.9% vs. 21.8%; *P* = 0.973) (**Table 1**).

**Table 1:** Characteristics of the analytical cohort according to prevalent CHD status.

Section	Characteristic	Overall (n=3964)	Non-CHD (n=2614)	Prevalent CHD (n=1350)	P value	Absolute SMD
Demographic and clinical	Age, years	62.7 ± 10.8	60.7 ± 10.5	66.7 ± 10.3	<0.001	0.58
	Male sex, n (%)	2,085 (52.6)	1,260 (48.2)	825 (61.1)	<0.001	0.26
	Body mass index, kg/m²	25.9 ± 3.8	25.6 ± 3.7	26.5 ± 3.9	<0.001	0.24
	Diabetes duration, years	9.2 ± 6.5	8.3 ± 6.1	10.9 ± 6.8	<0.001	0.40
	Hypertension, n (%)	2,140 (54.0)	1,210 (46.3)	930 (68.9)	<0.001	0.47
	Systolic blood pressure, mmHg	133.9 ± 16.2	131.8 ± 15.9	137.8 ± 15.9	<0.001	0.38
	Diastolic blood pressure, mmHg	79.3 ± 9.9	78.9 ± 9.9	80.0 ± 10.0	0.001	0.11
	Dyslipidemia, n (%)	1,875 (47.3)	1,120 (42.8)	755 (55.9)	<0.001	0.26
	Smoking status: Never, n (%)	2,355 (59.4)	1,639 (62.7)	716 (53.0)	<0.001	0.20
	Smoking status: Former, n (%)	599 (15.1)	360 (13.8)	239 (17.7)		0.11
	Smoking status: Current, n (%)	1,010 (25.5)	615 (23.5)	395 (29.3)		0.13
	Current drinking, n (%)	865 (21.8)	570 (21.8)	295 (21.9)	0.973	0.00
Glycemic and renal	HbA1c, %	7.9 ± 1.6	7.8 ± 1.5	8.1 ± 1.7	<0.001	0.19
	Fasting glucose, mmol/L	8.6 ± 2.5	8.5 ± 2.4	8.8 ± 2.6	<0.001	0.12
	Serum creatinine, μmol/L	80.9 ± 24.5	79.0 ± 22.7	84.6 ± 27.4	<0.001	0.22
	eGFR (CKD-EPI 2021), mL/min/1.73 m²	84.3 ± 18.6	86.1 ± 17.8	80.8 ± 19.7	<0.001	0.28
Conventional and derived lipids	Total cholesterol, mmol/L	4.9 ± 0.9	4.8 ± 0.9	5.0 ± 1.0	<0.001	0.23
	Triglycerides, mmol/L	1.90 (1.37–2.67)	1.81 (1.30–2.56)	2.08 (1.51–2.89)	<0.001	0.32
	HDL-C, mmol/L	1.10 ± 0.33	1.13 ± 0.33	1.05 ± 0.31	<0.001	0.25
	LDL-C, mmol/L	2.8 ± 0.8	2.7 ± 0.8	2.9 ± 0.9	<0.001	0.23
	Non-HDL-C, mmol/L	3.75 (3.04–4.51)	3.67 (3.00–4.35)	3.95 (3.25–4.70)	<0.001	0.24
	TG/HDL-C	1.80 (1.15–2.82)	1.68 (1.08–2.55)	2.05 (1.35–3.20)	<0.001	0.38
	Atherogenic index of plasma	0.25 (0.06–0.45)	0.23 (0.03–0.41)	0.31 (0.13–0.51)	<0.001	0.38
Hematologic and inflammatory	White blood cell count, ×10^9^/L	6.86 (5.85–8.11)	6.85 (5.85–8.03)	6.87 (5.84–8.30)	0.135	0.06
	Neutrophils, ×10^9^/L	4.26 (3.50–5.18)	4.17 (3.45–5.02)	4.44 (3.62–5.55)	<0.001	0.24
	Lymphocytes, ×10^9^/L	1.85 (1.36–2.45)	1.92 (1.43–2.54)	1.70 (1.24–2.28)	<0.001	0.29
	Monocytes, ×10^9^/L	0.43 (0.35–0.52)	0.42 (0.35–0.51)	0.43 (0.36–0.52)	0.001	0.11
	Platelets, ×10^9^/L	242 (201–292)	241 (200–290)	244 (203–298)	0.133	0.06
	High-sensitivity C-reactive protein, mg/L	2.05 (0.88–4.59)	1.80 (0.80–4.00)	2.60 (1.20–5.50)	<0.001	0.33
	NLR	2.31 (1.66–3.29)	2.17 (1.58–3.03)	2.62 (1.89–3.73)	<0.001	0.43
	PLR	131 (97–178)	125 (94–170)	144 (106–193)	<0.001	0.33
	LMR	4.29 (3.10–5.82)	4.54 (3.33–6.08)	3.85 (2.82–5.28)	<0.001	0.37
	SIRI	0.99 (0.69–1.43)	0.93 (0.65–1.33)	1.15 (0.81–1.66)	<0.001	0.47
	SII, ×10^9^/L	559 (401–799)	522 (378–734)	649 (454–899)	<0.001	0.47
	PIV	241 (156–364)	222 (145–336)	285 (187–429)	<0.001	0.45
Medication use	Any glucose-lowering medication, n (%)	3,542 (89.4)	2,300 (88.0)	1,242 (92.0)	<0.001	0.13
	Antihypertensive medication, n (%)	1,922 (48.5)	1,072 (41.0)	850 (63.0)	<0.001	0.45
	Lipid-lowering medication, n (%)	1,803 (45.5)	993 (38.0)	810 (60.0)	<0.001	0.45
	Insulin, n (%)	1,364 (34.4)	810 (31.0)	554 (41.0)	<0.001	0.21
	Metformin, n (%)	2,364 (59.6)	1,621 (62.0)	743 (55.0)	<0.001	0.14
	SGLT2 inhibitor, n (%)	594 (15.0)	392 (15.0)	202 (15.0)	0.978	0.00
	GLP-1 receptor agonist, n (%)	317 (8.0)	209 (8.0)	108 (8.0)	0.996	0.00
	Antiplatelet medication, n (%)	1,444 (36.4)	418 (16.0)	1,026 (76.0)	<0.001	1.51
	Systemic glucocorticoid, n (%)	59 (1.5)	39 (1.5)	20 (1.5)	0.979	0.00

Values are mean ± SD, median (interquartile range), or n (%). P values were calculated using Welch's t test, Wilcoxon rank-sum test, or chi-square/Fisher exact test, as appropriate. Absolute standardized mean differences (SMDs) are provided to describe group imbalance; skewed biomarkers were evaluated on the transformed scale. SII is expressed as ×10^9^/L. eGFR was calculated using the 2021 CKD-EPI equation.

Participants with CHD had higher HbA1c, fasting glucose, serum creatinine, total cholesterol, triglycerides, LDL-C, non-HDL-C, TG/HDL-C, and AIP, together with lower eGFR and HDL-C. The largest lipid-related differences were observed for TG/HDL-C [2.05 (1.35–3.20) vs. 1.68 (1.08–2.55); absolute SMD = 0.38] and AIP [0.31 (0.13–0.51) vs. 0.23 (0.03–0.41); absolute SMD = 0.38]. Inflammatory indices also differed between groups: participants with CHD had higher SII [649 (454–899) vs. 522 (378–734) ×10^9^/L; absolute SMD = 0.47], SIRI [1.15 (0.81–1.66) vs. 0.93 (0.65–1.33); absolute SMD = 0.47], PIV [285 (187–429) vs. 222 (145–336); absolute SMD = 0.45], NLR [2.62 (1.89–3.73) vs. 2.17 (1.58–3.03); absolute SMD = 0.43], PLR, and hs-CRP, whereas LMR was lower [3.85 (2.82–5.28) vs. 4.54 (3.33–6.08); absolute SMD = 0.37]. Total white blood cell and platelet counts did not differ significantly between groups.

Medication use differed substantially according to CHD status. Antiplatelet medication use was markedly more frequent among participants with CHD than among those without CHD (76.0% vs. 16.0%; absolute SMD = 1.51). Antihypertensive and lipid-lowering medication use were also more common in the CHD group (63.0% vs. 41.0% and 60.0% vs. 38.0%, respectively; absolute SMD = 0.45 for both). Participants with CHD more frequently used any glucose-lowering medication (92.0% vs. 88.0%) and insulin (41.0% vs. 31.0%) but less frequently used metformin (55.0% vs. 62.0%). Use of sodium-glucose cotransporter-2 inhibitors, glucagon-like peptide-1 receptor agonists, and systemic glucocorticoids was similar between groups.

### 3.3 Quartile-specific and standardized continuous associations with prevalent CHD

Unadjusted CHD prevalence generally increased across higher quartiles of the inflammatory indices. The prevalence increased from 22.8% in Q1 to 47.1% in Q4 for SII, from 23.0% to 45.7% for PIV, from 22.2% to 46.9% for SIRI, from 22.7% to 46.4% for NLR, and from 25.8% to 42.7% for PLR. Conversely, CHD prevalence decreased across LMR quartiles, from 44.0% in Q1 to 25.0% in Q4. AIP and TG/HDL-C had identical quartile allocations and prevalence patterns, increasing from 24.9% to 43.7%. For non-HDL-C, prevalence decreased slightly from Q1 to Q2 (30.2% to 29.1%) before increasing to 42.1% in Q4 (**Figure 2A**).

**Figure 2:**
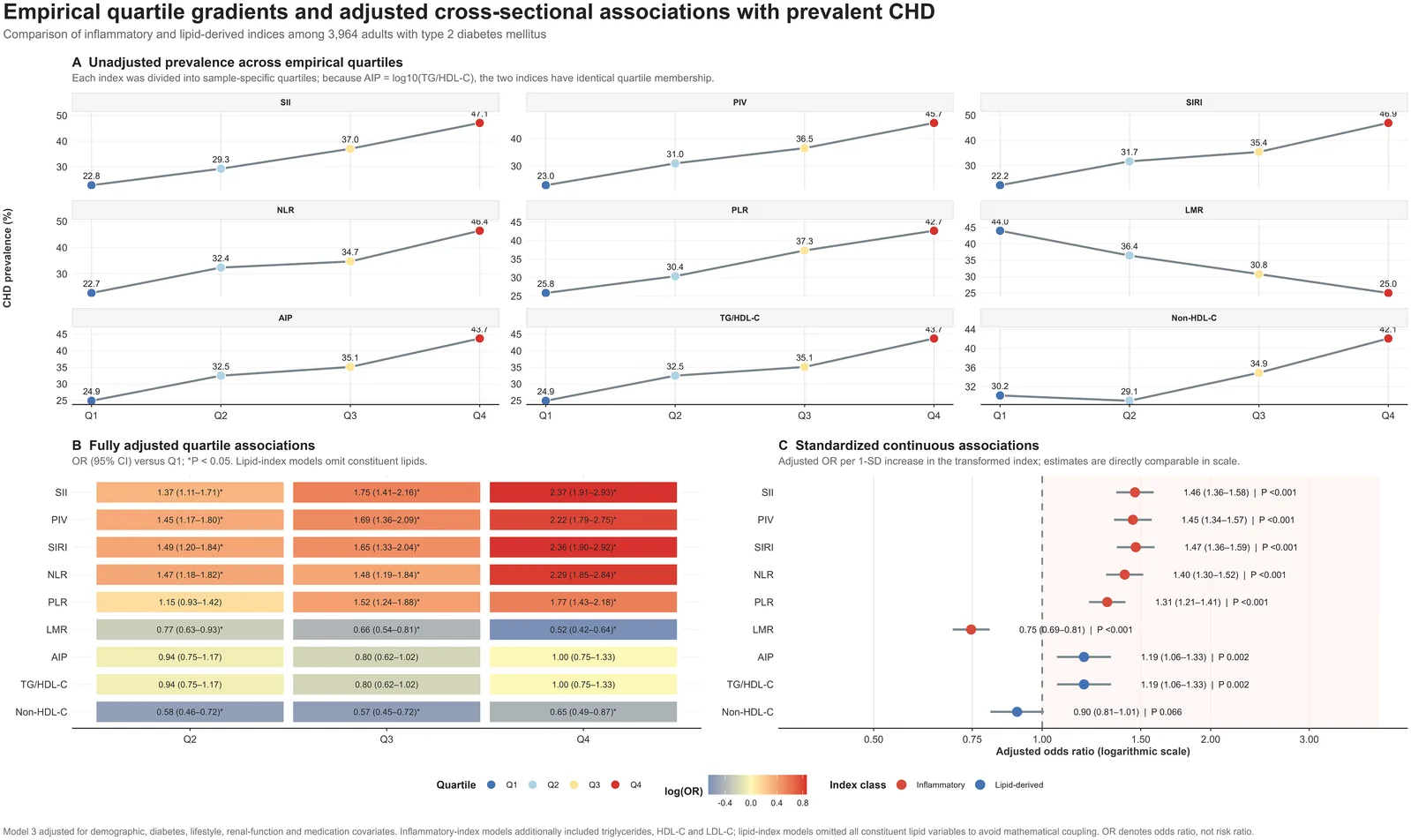
Quartile gradients and adjusted cross-sectional associations with prevalent coronary heart disease. **(A)** Unadjusted CHD prevalence across empirical quartiles. **(B)** Model 3 ORs (95% CIs) for Q2–Q4 versus Q1. **(C)** Model 3 ORs per 1-SD increase in the transformed index. Domain-specific Model 3 covariates were used, and lipid-index models omitted constituent lipid variables. AIP and TG/HDL-C used identical rankings. ORs describe odds of prevalent CHD, not risk ratios. CI, confidence interval; OR, odds ratio.

After domain-specific multivariable adjustment, all six inflammatory indices remained associated with prevalent CHD. Compared with Q1, the adjusted ORs for Q4 were 2.37 (95% CI, 1.91–2.93) for SII, 2.36 (95% CI, 1.90–2.92) for SIRI, 2.29 (95% CI, 1.85–2.84) for NLR, 2.22 (95% CI, 1.79–2.75) for PIV, and 1.77 (95% CI, 1.43–2.18) for PLR, with *P* for trend <0.001 for each index. LMR showed an inverse association, with an OR of 0.52 (95% CI, 0.42–0.64) for Q4 versus Q1 (*P* for trend <0.001). In the lipid-derived models that omitted constituent lipid measurements, neither AIP nor TG/HDL-C showed a graded quartile association; the Q4 OR was 1.00 (95% CI, 0.75–1.33), with *P* for trend = 0.703 for both. Higher quartiles of non-HDL-C were inversely associated with prevalent CHD after adjustment, with a Q4 OR of 0.65 (95% CI, 0.49–0.87; *P* for trend = 0.008) (**Figure 2B**; **Table 2**). Complete quartile-specific estimates across Models 1–3 are provided in **Supplementary Table S3** and **Supplementary Figure 1A**.

**Table 2:** Fully adjusted quartile-specific and standardized continuous associations with prevalent CHD.

Domain	Index	Q1 cases / n	Q2 OR (95% CI)	Q3 OR (95% CI)	Q4 OR (95% CI)	P for trend	Continuous OR per 1-SD (95% CI)	P continuous
Inflammatory	SII	226 / 991	1.37 (1.11–1.71)	1.75 (1.41–2.16)	2.37 (1.91–2.93)	<0.001	1.46 (1.36–1.58)	<0.001
	PIV	228 / 991	1.45 (1.17–1.80)	1.69 (1.36–2.09)	2.22 (1.79–2.75)	<0.001	1.45 (1.34–1.57)	<0.001
	SIRI	220 / 991	1.49 (1.20–1.84)	1.65 (1.33–2.04)	2.36 (1.90–2.92)	<0.001	1.47 (1.36–1.59)	<0.001
	NLR	225 / 991	1.47 (1.18–1.82)	1.48 (1.19–1.84)	2.29 (1.85–2.84)	<0.001	1.40 (1.30–1.52)	<0.001
	PLR	256 / 991	1.15 (0.93–1.42)	1.52 (1.24–1.88)	1.77 (1.43–2.18)	<0.001	1.31 (1.21–1.41)	<0.001
	LMR	436 / 991	0.77 (0.63–0.93)	0.66 (0.54–0.81)	0.52 (0.42–0.64)	<0.001	0.75 (0.69–0.81)	<0.001
Lipid-derived	AIP	247 / 991	0.94 (0.75–1.17)	0.80 (0.62–1.02)	1.00 (0.75–1.33)	0.703	1.19 (1.06–1.33)	0.002
	TG/HDL-C	247 / 991	0.94 (0.75–1.17)	0.80 (0.62–1.02)	1.00 (0.75–1.33)	0.703	1.19 (1.06–1.33)	0.002
	Non-HDL-C	299 / 991	0.58 (0.46–0.72)	0.57 (0.45–0.72)	0.65 (0.49–0.87)	0.008	0.90 (0.81–1.01)	0.066

Odds ratios (ORs) compare Q2–Q4 with Q1 and are not risk ratios. Continuous estimates are ORs per 1-SD increase in the transformed index. Model 3 adjusted for age, sex, body mass index, diabetes duration, hypertension, smoking status, current drinking, HbA1c, fasting glucose, eGFR, and glucose-lowering, antihypertensive and lipid-lowering medication use. Inflammatory-index models additionally included triglycerides, HDL-C and LDL-C. Models for AIP, TG/HDL-C and non-HDL-C omitted constituent lipids to avoid mathematical coupling. AIP and TG/HDL-C are mathematically monotonic and therefore do not represent independent information. P values are nominal and were not adjusted for multiplicity.

On the standardized continuous scale, each 1-SD increase in the transformed inflammatory indices was associated with higher odds of prevalent CHD for SIRI (OR, 1.47; 95% CI, 1.36–1.59), SII (OR, 1.46; 95% CI, 1.36–1.58), PIV (OR, 1.45; 95% CI, 1.34–1.57), NLR (OR, 1.40; 95% CI, 1.30–1.52), and PLR (OR, 1.31; 95% CI, 1.21–1.41), whereas LMR was inversely associated (OR, 0.75; 95% CI, 0.69–0.81); all nominal *P* values were <0.001. AIP and TG/HDL-C produced identical continuous estimates (OR, 1.19; 95% CI, 1.06–1.33; *P* = 0.002), although their quartile associations were non-monotonic. The continuous association for non-HDL-C did not reach nominal statistical significance (OR, 0.90; 95% CI, 0.81–1.01; *P* = 0.066) (**Figure 2C**; **Table 2**). Sequential continuous estimates are reported in **Supplementary Table S4** and **Supplementary Figure 1B**.

### 3.4 Correlation structure and dose-response relationships

The inflammatory indices were substantially correlated, consistent with their shared hematologic components. The strongest positive correlations were observed between PIV and SIRI (Spearman ρ = 0.85), SII and PIV (ρ = 0.84), SII and NLR (ρ = 0.80), and SIRI and NLR (ρ = 0.80). SII was also correlated with PLR (ρ = 0.76) and SIRI (ρ = 0.75). LMR showed inverse correlations with SIRI (ρ = −0.79), PLR (ρ = −0.68), PIV (ρ = −0.65), NLR (ρ = −0.59), and SII (ρ = −0.54). hs-CRP was positively correlated with SII and SIRI (ρ = 0.68 for both), PIV (ρ = 0.67), NLR (ρ = 0.60), and PLR (ρ = 0.57), and inversely correlated with LMR (ρ = −0.59). Correlations between the inflammatory and lipid-derived indices were weaker, with absolute coefficients ranging from 0.20 to 0.26. AIP and TG/HDL-C were perfectly rank-correlated (ρ = 1.00) because AIP is a monotonic transformation of TG/HDL-C; both were moderately correlated with non-HDL-C (ρ = 0.62) (**Figure 3A**; **Supplementary Table S6**).

**Figure 3:**
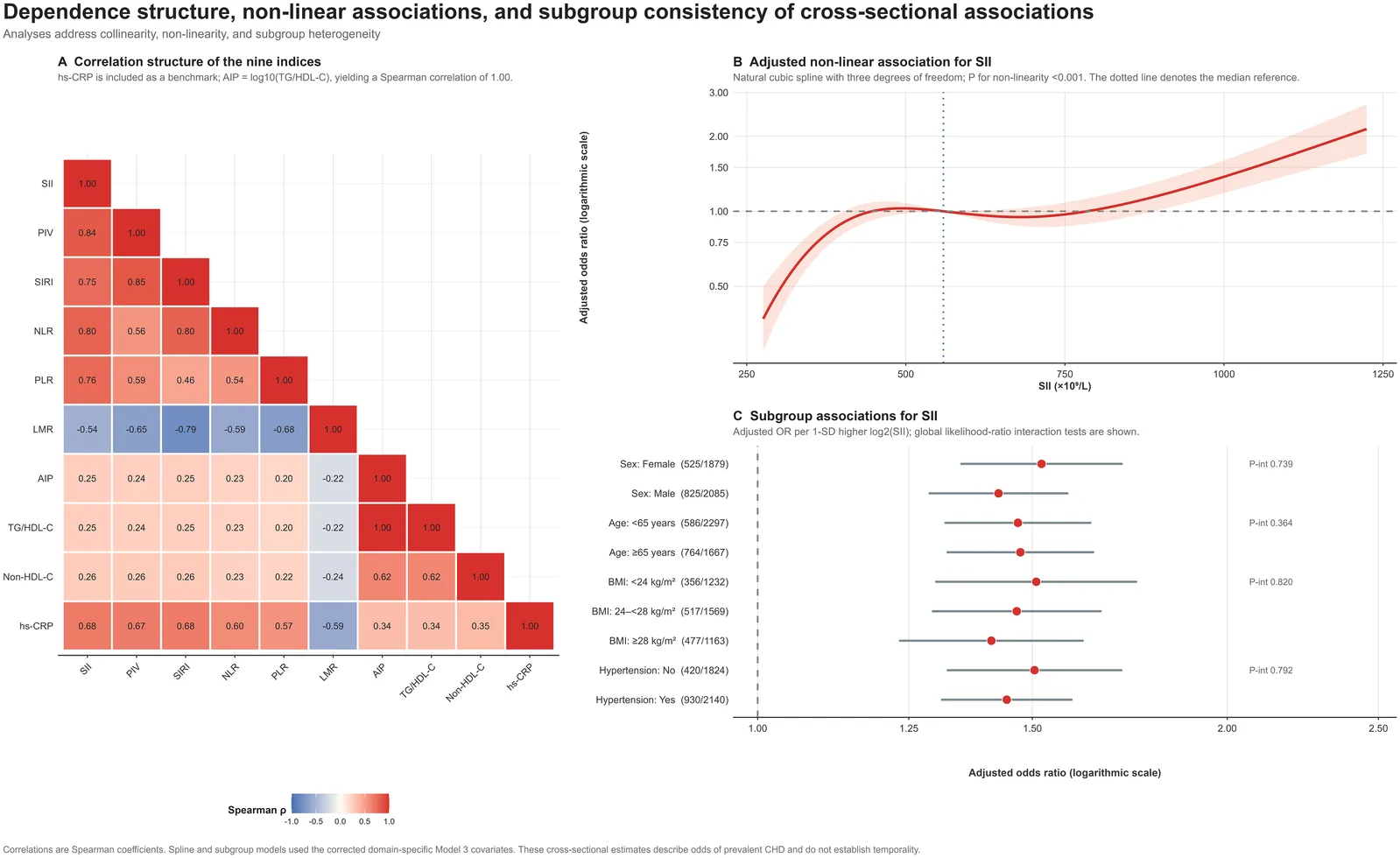
Correlation structure, dose-response association, and subgroup analyses. (**A**) Spearman correlation matrix of the nine indices and hs-CRP. **(B)** Adjusted natural-cubic-spline association between SII and prevalent CHD, with the median as reference and shading indicating the 95% CI. **(C)** Adjusted ORs per 1-SD increase in log_2_-transformed SII across the examined subgroups; global likelihood-ratio interaction *P* values are shown. CHD, coronary heart disease; hs-CRP, high-sensitivity C-reactive protein; OR, odds ratio; SII, systemic immune-inflammation index.

Adjusted spline analysis showed a nonlinear association between SII and prevalent CHD (*P* for nonlinearity <0.001). Relative to the median SII value of 559 ×10^9^/L, the estimated odds remained close to the reference across the middle of the distribution but increased progressively at higher SII values, with wider confidence intervals toward the distributional extremes (**Figure 3B**). Evidence of nonlinearity was also observed for PIV, SIRI, NLR, LMR, AIP, TG/HDL-C, and non-HDL-C (all *P* for nonlinearity <0.001) and for PLR (*P* for nonlinearity = 0.015). The inflammatory indices generally showed increasing odds in their upper ranges, whereas LMR showed a broadly inverse pattern. AIP and TG/HDL-C displayed identical non-monotonic curves, and non-HDL-C also showed a non-monotonic relationship, consistent with the differences between their quartile-based and continuous estimates (**Supplementary Table S8**; **Supplementary Figure 2A**).

### 3.5 Subgroup and sensitivity analyses

The positive association between SII and prevalent CHD was observed across all examined subgroups (**Figure 3C**; **Supplementary Table S5A**). The adjusted OR per 1-SD increase in log_2_(SII) was 1.52 (95% CI, 1.35–1.71) in women and 1.43 (95% CI, 1.29–1.58) in men (*P* for interaction = 0.739). Corresponding ORs were 1.47 (95% CI, 1.32–1.64) in participants aged <65 years and 1.47 (95% CI, 1.32–1.64) in those aged ≥65 years (*P* for interaction = 0.364). Across BMI categories, the ORs were 1.51 (95% CI, 1.30–1.75) for BMI <24 kg/m², 1.47 (95% CI, 1.29–1.66) for BMI 24–<28 kg/m², and 1.41 (95% CI, 1.23–1.62) for BMI ≥28 kg/m² (*P* for interaction = 0.820). The ORs were 1.50 (95% CI, 1.32–1.71) in participants without hypertension and 1.44 (95% CI, 1.31–1.59) in those with hypertension (*P* for interaction = 0.792). All subgroup-specific associations had *P* < 0.001, and none of the interaction tests indicated statistically significant heterogeneity.

The association remained similar in the temporal and reverse-causation sensitivity analyses (**Supplementary Table S5B**; **Supplementary Figure 2C**). Compared with the overall-cohort estimate of 1.46 (95% CI, 1.36–1.58), the adjusted OR was 1.45 (95% CI, 1.34–1.57) after excluding 135 participants with acute coronary syndrome at the index encounter, 1.43 (95% CI, 1.32–1.56) after excluding 243 cases newly ascertained at the index encounter, and 1.44 (95% CI, 1.32–1.57) when the analysis was restricted to participants whose CHD had been documented at least 365 days before laboratory measurement. All sensitivity-analysis estimates had *P* < 0.001.

### 3.6 Internal validation, calibration, and decision-curve analysis

The common clinical base model had an apparent AUC of 0.733 and an optimism-corrected AUC of 0.729. After bootstrap correction, the largest increment relative to the base model was observed for SIRI (ΔAUC = +0.016), followed by SII and PIV (both ΔAUC = +0.015); all corresponding *P* values were <0.001. The corrected increments were +0.012 for NLR, + 0.010 for LMR, and +0.009 for PLR (all *P* < 0.001). A smaller but statistically significant increment was observed for hs-CRP (ΔAUC = +0.004; *P* = 0.018). In contrast, the increments associated with AIP and TG/HDL-C were not statistically significant (both ΔAUC = +0.002; *P* = 0.123), nor was that associated with non-HDL-C (ΔAUC = +0.000; *P* = 0.514). The location-shifted 95% CIs excluded zero for SIRI, SII, PIV, NLR, LMR, PLR, and hs-CRP but included zero for AIP, TG/HDL-C, and non-HDL-C (**Figure 4A**; **Table 3**).

**Figure 4:**
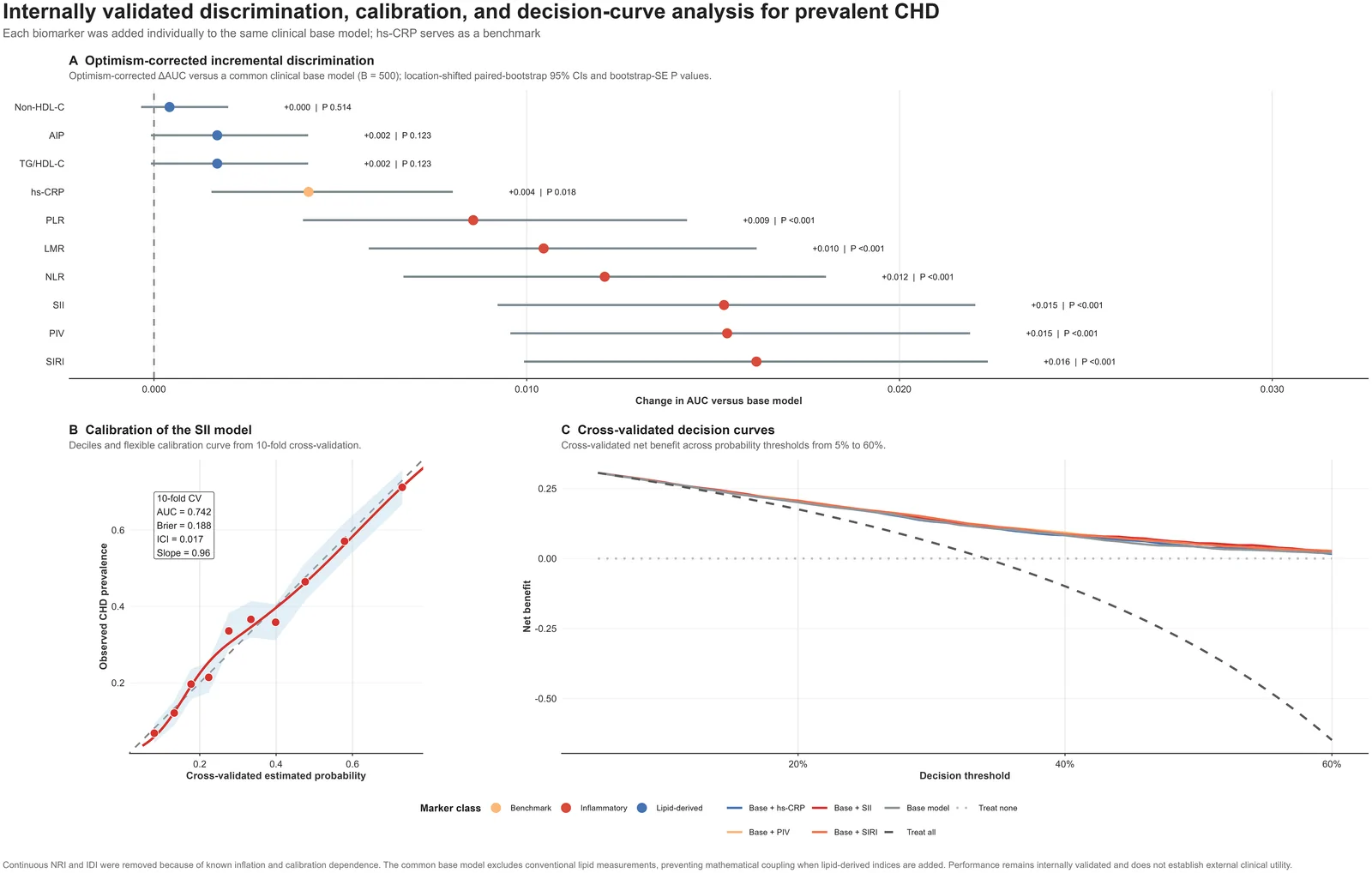
Internally validated incremental discrimination, calibration, and decision-curve analysis for prevalent coronary heart disease. (**A**) Optimism-corrected change in the area under the receiver-operating-characteristic curve (ΔAUC) after adding each marker separately to the common clinical base model. Estimates were obtained from 500 nonparametric participant-level paired bootstrap samples, using identical resampling indices for the base and marker-augmented models. Error bars represent location-shifted paired-bootstrap 95% confidence intervals; two-sided *P* values were calculated using a normal approximation based on the optimism-corrected ΔAUC and its paired-bootstrap standard error. **(B)** Calibration of the SII-augmented model based on 10-fold cross-validated predicted probabilities. **(C)** Cross-validated decision curves comparing the clinical base model with models augmented by SII, PIV, SIRI, or hs-CRP. These analyses represent internal validation and do not constitute external or prospective validation. AIP and TG/HDL-C overlap because AIP is a logarithmic transformation of TG/HDL-C; their identical estimates do not represent independent evidence. AIP, atherogenic index of plasma; AUC, area under the receiver-operating-characteristic curve; CHD, coronary heart disease; hs-CRP, high-sensitivity C-reactive protein; PIV, pan-immune-inflammation value; SII, systemic immune-inflammation index; SIRI, systemic inflammation response index; TG/HDL-C, triglyceride-to-high-density lipoprotein cholesterol ratio.

**Table 3:** Internally validated incremental discrimination relative to the common clinical base model.

Model	Marker class	Apparent AUC	Optimism-corrected AUC	Corrected ΔAUC (95% CI)	*P* vs base	SII minus comparator ΔAUC (95% CI)	*P* vs SII
Base model	Clinical	0.733	0.729	Reference	—	—	—
Base + SII	Inflammatory	0.748	0.744	+0.015 (0.009–0.022)	<0.001	Reference	—
Base + PIV	Inflammatory	0.749	0.744	+0.015 (0.010–0.022)	<0.001	+0.000 (−0.004–0.004)	0.964
Base + SIRI	Inflammatory	0.749	0.745	+0.016 (0.010–0.022)	<0.001	−0.001 (−0.006–0.004)	0.717
Base + NLR	Inflammatory	0.745	0.741	+0.012 (0.007–0.018)	<0.001	+0.003 (−0.001–0.007)	0.112
Base + PLR	Inflammatory	0.742	0.737	+0.009 (0.004–0.014)	<0.001	+0.007 (0.003–0.011)	0.002
Base + LMR	Inflammatory	0.744	0.739	+0.010 (0.006–0.016)	<0.001	+0.005 (−0.001–0.011)	0.106
Base + AIP	Lipid-derived	0.735	0.731	+0.002 (−0.000–0.004)	0.123	+0.014 (0.008–0.021)	<0.001
Base + TG/HDL-C	Lipid-derived	0.735	0.731	+0.002 (−0.000–0.004)	0.123	+0.014 (0.008–0.021)	<0.001
Base + Non-HDL-C	Lipid-derived	0.734	0.729	+0.000 (−0.000–0.002)	0.514	+0.015 (0.008–0.022)	<0.001
Base + hs-CRP	Benchmark	0.738	0.733	+0.004 (0.002–0.008)	0.018	+0.011 (0.006–0.016)	<0.001

AUC estimates were optimism-corrected using 500 nonparametric participant-level paired bootstrap samples. Identical resampling indices were used for the base and marker-augmented models. Location-shifted 95% confidence intervals were obtained by shifting the percentile limits of the bootstrap distribution of the apparent paired ΔAUC by the estimated mean optimism. Two-sided *P* values were calculated using a normal approximation based on the optimism-corrected ΔAUC and its paired-bootstrap standard error. The common clinical base model excluded conventional lipid measurements to prevent mathematical coupling when lipid-derived indices were added. Pairwise differences are reported as SII minus the comparator; positive values favor SII. These internally validated estimates do not constitute external prediction validation. All *P* values are nominal and should be interpreted with the effect estimates and confidence intervals rather than as a biomarker-ranking procedure.

In direct paired-bootstrap comparisons, the corrected AUC of the SII-augmented model did not differ significantly from those of the PIV-augmented model (SII minus PIV ΔAUC = +0.000; *P* = 0.964), SIRI-augmented model (ΔAUC = −0.001; *P* = 0.717), NLR-augmented model (ΔAUC = +0.003; *P* = 0.112), or LMR-augmented model (ΔAUC = +0.005; *P* = 0.106). The SII-augmented model had a higher corrected AUC than the PLR-augmented model (ΔAUC = +0.007; *P* = 0.002) and models augmented with hs-CRP (ΔAUC = +0.011), AIP (+0.014), TG/HDL-C (+0.014), or non-HDL-C (+0.015); the corresponding location-shifted 95% CIs excluded zero (all *P* < 0.001) (**Supplementary Figure 2B**; **Supplementary Table S7A**).

For the SII-augmented model, the apparent, optimism-corrected, and 10-fold cross-validated AUCs were 0.748, 0.744, and 0.742, respectively. Ten-fold cross-validation yielded a Brier score of 0.188, an integrated calibration index of 0.017, a calibration intercept of −0.001, and a calibration slope of 0.963. The bootstrap-corrected calibration slope and Brier score were 0.977 and 0.188, respectively (**Figure 4B**; **Supplementary Table S7B**). In the decision-curve analysis, the base and marker-augmented models showed closely overlapping net-benefit curves across the displayed threshold range, with only small differences among the SII-, PIV-, SIRI-, hs-CRP-, and base-model curves (**Figure 4C**).

## 4 Discussion

In this cross-sectional study of 3,964 adults with T2DM, the six CBC-derived inflammatory indices were consistently associated with the odds of prevalent CHD after multivariable adjustment, whereas the lipid-derived indices showed weaker and less consistent patterns. On the standardized continuous scale, SIRI, SII, and PIV yielded similar adjusted ORs, while substantial correlations among these indices indicated that they captured overlapping hematologic information. When each marker was added separately to the common non-lipid clinical base model, the largest optimism-corrected increment in AUC was observed for SIRI, followed closely by SII and PIV. Direct paired comparisons showed no statistically significant differences between the SII-augmented model and models augmented with PIV, SIRI, NLR, or LMR, further indicating that these correlated CBC-derived indices provided broadly similar discrimination. Moreover, the decision curves showed little separation among the base and marker-augmented models.

Our findings are consistent with observational evidence linking systemic inflammation to atherosclerosis and cardiovascular complications in populations with diabetes. Previous studies have reported associations of SII, SIRI, and NLR with coronary phenotypes or adverse cardiovascular outcomes, although the incremental information provided by these markers beyond established clinical factors has generally been modest (11–13). CBC-derived inflammatory indices have also been associated with cardiovascular or all-cause mortality in different high-risk populations (14, 15). Positive associations of NLR and SII with cardiovascular disease or mortality have been reported among individuals with diabetes or prediabetes (7, 16, 17). In parallel, AIP has been associated with the presence and angiographic severity of coronary artery disease in patients with T2DM (18). The present study extends this literature by comparing multiple inflammatory and lipid-derived indices within the same cohort, using a common standardized exposure scale and explicitly accounting for their dependence structure. Nevertheless, because the outcome was prevalent rather than incident CHD, our results should be interpreted as cross-sectional associations and not as evidence of prognostic prediction.

Several biological pathways may plausibly underlie the observed associations between CBC-derived inflammatory indices and prevalent CHD. Chronic vascular inflammation contributes to endothelial dysfunction, lipid accumulation, plaque formation, and plaque destabilization (19). In CANTOS, targeted inhibition of interleukin-1β with canakinumab reduced recurrent cardiovascular events independently of lipid-level lowering, supporting the broader clinical importance of inflammatory pathways in atherosclerotic disease (20). Reactive oxygen species contribute to endothelial dysfunction and vascular injury in atherosclerosis (21, 22). Platelets participate in leukocyte recruitment, thrombo-inflammation, and arterial thrombosis, while adaptive immune responses involving lymphocyte populations influence atherosclerotic inflammation and post-myocardial-infarction injury (23). SII and the related indices combine these cellular components in different mathematical forms and may therefore reflect multiple aspects of the inflammatory and immune milieu. These mechanisms provide biological plausibility but do not establish that elevations in the indices preceded or caused CHD in the present cohort.

The strong correlations among the inflammatory indices are important for their comparative interpretation. SII was highly correlated with PIV, NLR, PLR, and SIRI, while PIV and SIRI were also strongly correlated. Correspondingly, their standardized continuous ORs and optimism-corrected changes in AUC were similar. Direct location-shifted paired-bootstrap comparisons showed no statistically significant differences between the SII-augmented model and models augmented with PIV, SIRI, NLR, or LMR, despite small numerical differences in their corrected AUCs. Although the SII-augmented model had a higher corrected AUC than models augmented with PLR, hs-CRP, or the lipid-derived indices, these comparisons were exploratory, were not adjusted for multiple testing, and represented only modest absolute differences in discrimination. Collectively, these findings indicate that the CBC-derived indices largely capture overlapping information and that no single inflammatory index can be considered clearly superior on the basis of the present data.

The lipid-derived indices displayed a different and more complex pattern. AIP and TG/HDL-C are mathematically monotonic transformations of the same lipid ratio and consequently produced identical rankings, quartile allocations, and standardized continuous estimates; they should not be regarded as independent sources of information. Although their standardized continuous associations were nominally significant, neither index showed a graded adjusted quartile association, and spline analyses suggested nonlinearity. Non-HDL-C showed an inverse Q4-versus-Q1 association after adjustment, whereas its standardized continuous association did not reach nominal statistical significance. This discordance should not be interpreted as evidence that higher non-HDL-C protects against CHD. Established CHD and its treatment may alter lipid concentrations, particularly through the greater use of lipid-lowering therapy among participants with CHD, thereby introducing treatment-related confounding and reverse causation. The nonlinear and model-dependent findings for the lipid-derived indices therefore warrant cautious interpretation and prospective evaluation.

From a clinical perspective, CBC-derived inflammatory indices are inexpensive and readily available, but accessibility alone does not establish clinical usefulness. The internally validated improvements in discrimination were modest, and the cross-validated decision curves showed only small differences in net benefit between the base and marker-augmented models. In addition, the analyses evaluated discrimination of prevalent CHD rather than prediction of future cardiovascular events. Accordingly, the present findings do not support using SII or the other indices for cardiovascular risk stratification, selection of preventive treatment, or identification of patients requiring more intensive intervention. Their potential clinical role would need to be evaluated in prospective cohorts with a clearly defined prediction horizon, clinically relevant decision thresholds, and external validation.

This study has several strengths. It included a relatively large cohort of adults with T2DM and evaluated six inflammatory indices, three lipid-derived indices, and hs-CRP within the same analytical population. Domain-specific adjustment avoided conditioning the lipid-derived indices on their mathematical components. Standardized continuous estimates facilitated head-to-head comparison, while correlation analyses demonstrated the substantial dependence among the indices. The study also incorporated spline analyses, direct paired comparisons of discrimination, bootstrap optimism correction, cross-validated calibration, decision-curve analysis, and temporal sensitivity analyses.

Several limitations should be acknowledged. First, the cross-sectional design precludes establishing temporality or causality, and reverse causation cannot be excluded because established CHD, acute coronary events, and subsequent treatment may influence blood-cell counts and lipid concentrations. Although temporal sensitivity analyses yielded similar estimates, they cannot eliminate this possibility. Second, this was a single-center study conducted in a tertiary referral hospital, and the high CHD prevalence, complete-case selection, and differences between included and excluded participants may have introduced referral and selection bias, limiting generalizability. Third, the CHD definition combined diagnostic codes, previous myocardial infarction, revascularization, and imaging-confirmed stenosis, and independent outcome adjudication was unavailable, so some outcome misclassification may remain. Residual confounding from unmeasured clinical, lifestyle, and treatment-related factors is also possible. In addition, the common base model excluded conventional lipid measurements to avoid mathematical coupling with lipid-derived indices, which may affect comparisons with conventional clinical models. Finally, model performance was internally but not externally validated, and multiple exploratory analyses were conducted without multiplicity adjustment. Prospective multicenter studies with external validation are therefore required.

## 5 Conclusion

In this cross-sectional cohort of adults with T2DM, several CBC-derived inflammatory indices were associated with the odds of prevalent CHD, whereas lipid-derived indices showed weaker and nonlinear associations. SII, PIV, and SIRI provided similarly modest improvements in internally validated discrimination, with no consistent evidence that SII was superior. These findings are hypothesis-generating and do not establish temporality, prediction of incident CHD, or clinical utility. Prospective multicenter studies with external validation are required before these indices can be considered for cardiovascular risk prediction or clinical decision-making.

## Data Availability

The raw data supporting the conclusions of this article will be made available by the authors, without undue reservation.
