# Supplementary material for: Cross-sectional associations and incremental discrimination of inflammatory and lipid-derived indices for prevalent coronary heart disease in type 2 diabetes
Source: Front Endocrinol (Lausanne). 2026 Sep 24;17:1961170. doi: 10.3389/fendo.2026.1961170 (PMC13645562; doi:10.3389/fendo.2026.1961170)
Supplement: Supplementary file 1 [file SupplementaryFile1.docx]

**Table S1A. Mutually exclusive primary reasons for exclusion**

| **Primary exclusion reason** | **Number excluded** | **Percent of excluded** |
| --- | --- | --- |
| Incomplete clinical data | 310 | 41.0 |
| Acute infection | 120 | 15.9 |
| Severe renal or hepatic impairment | 110 | 14.6 |
| Autoimmune or hematologic disorder | 80 | 10.6 |
| Malignancy | 80 | 10.6 |
| Type 1 or gestational diabetes | 56 | 7.4 |

Exclusion reasons were assigned hierarchically so that each excluded record contributed to one primary category.

# Table S1B. Missingness of core variables in the source population

| **Variable** | **Missing n** | **Missing %** |
| --- | --- | --- |
| Age | 0 | 0.0 |
| Sex | 0 | 0.0 |
| Body mass index | 108 | 2.3 |
| Diabetes duration | 120 | 2.5 |
| Hypertension | 0 | 0.0 |
| Smoking status | 0 | 0.0 |
| HbA1c | 126 | 2.7 |
| Fasting glucose | 126 | 2.7 |
| eGFR (CKD-EPI 2021) | 135 | 2.9 |
| Total cholesterol | 121 | 2.6 |
| Triglycerides | 111 | 2.4 |
| HDL-C | 123 | 2.6 |
| LDL-C | 140 | 3.0 |
| Neutrophils | 117 | 2.5 |
| Lymphocytes | 127 | 2.7 |
| Monocytes | 123 | 2.6 |
| Platelets | 110 | 2.3 |
| hs-CRP | 118 | 2.5 |

Percentages use the complete source population as the denominator. Structurally inapplicable CHD dates among non-CHD participants are not treated as missing core data.

# Table S1C. Comparison of included and excluded records

| **Section** | **Characteristic** | **Excluded (n=756)** | **Included (n=3964)** | **P value** | **Absolute SMD** |
| --- | --- | --- | --- | --- | --- |
| **Demographic and clinical** | Age, years | 62.5 ± 10.8 | 62.7 ± 10.8 | 0.613 | 0.02 |
|  | Male sex, n (%) | 384 (50.8) | 2,085 (52.6) | 0.363 | 0.04 |
|  | Body mass index, kg/m² | 25.8 ± 3.8 | 25.9 ± 3.8 | 0.441 | 0.03 |
|  | Diabetes duration, years | 8.9 ± 6.5 | 9.2 ± 6.5 | 0.361 | 0.04 |
|  | Hypertension, n (%) | 403 (53.3) | 2,140 (54.0) | 0.731 | 0.01 |
|  | Smoking status: Never, n (%) | 452 (59.8) | 2,355 (59.4) | 0.375 | 0.01 |
|  | Smoking status: Former, n (%) | 126 (16.7) | 599 (15.1) |  | 0.04 |
|  | Smoking status: Current, n (%) | 178 (23.5) | 1,010 (25.5) |  | 0.04 |
| **Glycemic and renal** | HbA1c, % | 7.9 ± 1.6 | 7.9 ± 1.6 | 0.800 | 0.01 |
|  | eGFR (CKD-EPI 2021), mL/min/1.73 m² | 71.4 ± 31.7 | 84.3 ± 18.6 | <0.001 | 0.50 |
| **Conventional and derived lipids** | Triglycerides, mmol/L | 1.95 (1.38–2.70) | 1.90 (1.37–2.67) | 0.673 | 0.01 |
|  | HDL-C, mmol/L | 1.12 ± 0.33 | 1.10 ± 0.33 | 0.327 | 0.04 |
| **Hematologic and inflammatory** | High-sensitivity C-reactive protein, mg/L | 3.03 (1.04–8.65) | 2.05 (0.88–4.59) | <0.001 | 0.46 |
|  | SII, ×10⁹/L | 558 (400–773) | 559 (401–799) | 0.644 | 0.02 |

Summaries use available observations. This descriptive comparison assesses the potential for selection bias arising from complete-case inclusion.

# Table S2A. Mutually exclusive primary basis for CHD ascertainment

| **Mutually exclusive primary criterion** | **Number of cases** | **Percent of CHD cases** |
| --- | --- | --- |
| Inpatient ICD-10 I20-I25 | 389 | 28.8 |
| Imaging stenosis >=50% | 273 | 20.2 |
| Prior PCI/CABG | 239 | 17.7 |
| Two outpatient ICD-10 records | 235 | 17.4 |
| Prior myocardial infarction | 214 | 15.9 |

Primary criteria are mutually exclusive and sum to 1,350 prevalent CHD cases.

# Table S2B. Timing of CHD ascertainment relative to biomarker measurement

| **CHD status relative to index encounter** | **Number of cases** | **Percent of CHD cases** |
| --- | --- | --- |
| Newly ascertained at index encounter | 243 | 18.0 |
| Previously documented CHD | 1,107 | 82.0 |

Biomarkers were measured at the index encounter. Temporal ambiguity remains for CHD newly ascertained at that encounter.

# Table S2C. Diagnostic evidence and clinical composition of CHD cases

| **Diagnostic evidence or clinical feature** | **Number of CHD cases** | **Percent of CHD cases** |
| --- | --- | --- |
| Inpatient ICD-10 I20–I25 | 713 | 52.8 |
| At least two outpatient ICD-10 records | 489 | 36.2 |
| Prior myocardial infarction | 473 | 35.0 |
| Prior PCI | 539 | 39.9 |
| Prior CABG | 95 | 7.0 |
| Imaging-confirmed coronary stenosis ≥50% | 665 | 49.3 |
| Acute coronary syndrome at index | 135 | 10.0 |

Diagnostic evidence categories may overlap and therefore do not sum to 100%.

# Table S3. Complete quartile- and model-specific associations with prevalent CHD

| **Index** | **Quartile** | **Cutpoints** | **Cases / n** | **Model 1** | **Model 2** | **Model 3** |
| --- | --- | --- | --- | --- | --- | --- |
| SII | Q1 | ≤401 | 226 / 991 | 1.00 (reference) | 1.00 (reference) | 1.00 (reference) |
| SII | Q2 | 401–559 | 290 / 991 | 1.40 (1.14–1.71)* | 1.30 (1.06–1.60)* | 1.37 (1.11–1.71)* |
| SII | Q3 | 559–798 | 367 / 991 | 1.99 (1.64–2.42)* | 1.71 (1.40–2.10)* | 1.75 (1.41–2.16)* |
| SII | Q4 | ≥800 | 467 / 991 | 3.02 (2.49–3.66)* | 2.26 (1.84–2.76)* | 2.37 (1.91–2.93)* |
| SII | P for trend | — | — | <0.001 | <0.001 | <0.001 |
| PIV | Q1 | ≤156 | 228 / 991 | 1.00 (reference) | 1.00 (reference) | 1.00 (reference) |
| PIV | Q2 | 156–241 | 307 / 991 | 1.50 (1.23–1.83)* | 1.39 (1.14–1.71)* | 1.45 (1.17–1.80)* |
| PIV | Q3 | 241–364 | 362 / 991 | 1.93 (1.58–2.34)* | 1.64 (1.34–2.01)* | 1.69 (1.36–2.09)* |
| PIV | Q4 | ≥364 | 453 / 991 | 2.82 (2.32–3.42)* | 2.08 (1.70–2.55)* | 2.22 (1.79–2.75)* |
| PIV | P for trend | — | — | <0.001 | <0.001 | <0.001 |
| SIRI | Q1 | ≤0.69 | 220 / 991 | 1.00 (reference) | 1.00 (reference) | 1.00 (reference) |
| SIRI | Q2 | 0.69–0.99 | 314 / 991 | 1.63 (1.33–1.99)* | 1.49 (1.21–1.83)* | 1.49 (1.20–1.84)* |
| SIRI | Q3 | 0.99–1.43 | 351 / 991 | 1.92 (1.58–2.34)* | 1.62 (1.32–1.98)* | 1.65 (1.33–2.04)* |
| SIRI | Q4 | ≥1.43 | 465 / 991 | 3.10 (2.55–3.77)* | 2.29 (1.87–2.81)* | 2.36 (1.90–2.92)* |
| SIRI | P for trend | — | — | <0.001 | <0.001 | <0.001 |
| NLR | Q1 | ≤1.66 | 225 / 991 | 1.00 (reference) | 1.00 (reference) | 1.00 (reference) |
| NLR | Q2 | 1.66–2.31 | 321 / 991 | 1.63 (1.34–1.99)* | 1.46 (1.18–1.79)* | 1.47 (1.18–1.82)* |
| NLR | Q3 | 2.31–3.29 | 344 / 991 | 1.81 (1.48–2.21)* | 1.53 (1.25–1.88)* | 1.48 (1.19–1.84)* |
| NLR | Q4 | ≥3.29 | 460 / 991 | 2.95 (2.43–3.58)* | 2.22 (1.81–2.72)* | 2.29 (1.85–2.84)* |
| NLR | P for trend | — | — | <0.001 | <0.001 | <0.001 |
| PLR | Q1 | ≤97 | 256 / 991 | 1.00 (reference) | 1.00 (reference) | 1.00 (reference) |
| PLR | Q2 | 97–131 | 301 / 991 | 1.25 (1.03–1.52)* | 1.13 (0.92–1.38) | 1.15 (0.93–1.42) |
| PLR | Q3 | 131–178 | 370 / 991 | 1.71 (1.41–2.07)* | 1.44 (1.18–1.76)* | 1.52 (1.24–1.88)* |
| PLR | Q4 | ≥178 | 423 / 991 | 2.14 (1.77–2.59)* | 1.68 (1.38–2.05)* | 1.77 (1.43–2.18)* |
| PLR | P for trend | — | — | <0.001 | <0.001 | <0.001 |
| LMR | Q1 | ≤3.10 | 436 / 991 | 1.00 (reference) | 1.00 (reference) | 1.00 (reference) |
| LMR | Q2 | 3.10–4.29 | 361 / 991 | 0.73 (0.61–0.87)* | 0.81 (0.67–0.98)* | 0.77 (0.63–0.93)* |
| LMR | Q3 | 4.29–5.82 | 305 / 991 | 0.57 (0.47–0.68)* | 0.67 (0.56–0.82)* | 0.66 (0.54–0.81)* |
| LMR | Q4 | ≥5.82 | 248 / 991 | 0.42 (0.35–0.51)* | 0.55 (0.45–0.68)* | 0.52 (0.42–0.64)* |
| LMR | P for trend | — | — | <0.001 | <0.001 | <0.001 |
| AIP | Q1 | ≤0.06 | 247 / 991 | 1.00 (reference) | 1.00 (reference) | 1.00 (reference) |
| AIP | Q2 | 0.06–0.25 | 322 / 991 | 1.45 (1.19–1.76)* | 0.99 (0.80–1.22) | 0.94 (0.75–1.17) |
| AIP | Q3 | 0.25–0.45 | 348 / 991 | 1.63 (1.34–1.98)* | 0.86 (0.69–1.07) | 0.80 (0.62–1.02) |
| AIP | Q4 | ≥0.45 | 433 / 991 | 2.34 (1.93–2.83)* | 0.84 (0.66–1.08) | 1.00 (0.75–1.33) |
| AIP | P for trend | — | — | <0.001 | 0.099 | 0.703 |
| TG/HDL-C | Q1 | ≤1.15 | 247 / 991 | 1.00 (reference) | 1.00 (reference) | 1.00 (reference) |
| TG/HDL-C | Q2 | 1.15–1.80 | 322 / 991 | 1.45 (1.19–1.76)* | 0.99 (0.80–1.22) | 0.94 (0.75–1.17) |
| TG/HDL-C | Q3 | 1.80–2.82 | 348 / 991 | 1.63 (1.34–1.98)* | 0.86 (0.69–1.07) | 0.80 (0.62–1.02) |
| TG/HDL-C | Q4 | ≥2.82 | 433 / 991 | 2.34 (1.93–2.83)* | 0.84 (0.66–1.08) | 1.00 (0.75–1.33) |
| TG/HDL-C | P for trend | — | — | <0.001 | 0.099 | 0.703 |
| Non-HDL-C | Q1 | ≤3.04 | 299 / 991 | 1.00 (reference) | 1.00 (reference) | 1.00 (reference) |
| Non-HDL-C | Q2 | 3.04–3.75 | 288 / 991 | 0.95 (0.78–1.15) | 0.61 (0.49–0.75)* | 0.58 (0.46–0.72)* |
| Non-HDL-C | Q3 | 3.75–4.51 | 346 / 991 | 1.24 (1.03–1.50)* | 0.63 (0.50–0.78)* | 0.57 (0.45–0.72)* |
| Non-HDL-C | Q4 | ≥4.51 | 417 / 991 | 1.68 (1.40–2.02)* | 0.54 (0.43–0.69)* | 0.65 (0.49–0.87)* |
| Non-HDL-C | P for trend | — | — | <0.001 | <0.001 | 0.008 |

Values are OR (95% CI) for Q2–Q4 versus Q1; *P<0.05. Model 1 was unadjusted; Model 2 adjusted for age and sex; Model 3 used the domain-specific covariate sets defined in Table 2. Quartiles are empirical sample-specific groups. P values are nominal and were not adjusted for multiplicity.

# Table S4. Standardized continuous associations across sequential models

| **Domain** | **Index** | **Model 1** | **Model 2** | **Model 3** |
| --- | --- | --- | --- | --- |
| **Inflammatory** | SII | 1.61 (1.50–1.72) \| P <0.001 | 1.44 (1.34–1.55) \| P <0.001 | 1.46 (1.36–1.58) \| P <0.001 |
|  | PIV | 1.58 (1.48–1.70) \| P <0.001 | 1.42 (1.32–1.52) \| P <0.001 | 1.45 (1.34–1.57) \| P <0.001 |
|  | SIRI | 1.62 (1.51–1.73) \| P <0.001 | 1.45 (1.34–1.55) \| P <0.001 | 1.47 (1.36–1.59) \| P <0.001 |
|  | NLR | 1.54 (1.44–1.65) \| P <0.001 | 1.39 (1.30–1.50) \| P <0.001 | 1.40 (1.30–1.52) \| P <0.001 |
|  | PLR | 1.40 (1.31–1.50) \| P <0.001 | 1.27 (1.19–1.37) \| P <0.001 | 1.31 (1.21–1.41) \| P <0.001 |
|  | LMR | 0.69 (0.64–0.74) \| P <0.001 | 0.76 (0.71–0.82) \| P <0.001 | 0.75 (0.69–0.81) \| P <0.001 |
| **Lipid-derived** | AIP | 1.46 (1.37–1.57) \| P <0.001 | 1.04 (0.95–1.14) \| P 0.423 | 1.19 (1.06–1.33) \| P 0.002 |
|  | TG/HDL-C | 1.46 (1.37–1.57) \| P <0.001 | 1.04 (0.95–1.14) \| P 0.423 | 1.19 (1.06–1.33) \| P 0.002 |
|  | Non-HDL-C | 1.28 (1.20–1.38) \| P <0.001 | 0.84 (0.76–0.92) \| P <0.001 | 0.90 (0.81–1.01) \| P 0.066 |

Values are OR (95% CI) per 1-SD increase in the transformed index. All indices except AIP were log2-transformed before standardization. P values are nominal and were not adjusted for multiplicity.

# Table S5A. Subgroup associations for SII

| **Subgroup level** | **CHD cases / n** | **Adjusted OR per 1-SD log2(SII) (95% CI)** | **P value** | **P for interaction** |
| --- | --- | --- | --- | --- |
| Sex: Female | 525 / 1879 | 1.52 (1.35–1.71) | <0.001 | 0.739 |
| Sex: Male | 825 / 2085 | 1.43 (1.29–1.58) | <0.001 |  |
| Age: <65 years | 586 / 2297 | 1.47 (1.32–1.64) | <0.001 | 0.364 |
| Age: ≥65 years | 764 / 1667 | 1.47 (1.32–1.64) | <0.001 |  |
| BMI: <24 kg/m² | 356 / 1232 | 1.51 (1.30–1.75) | <0.001 | 0.820 |
| BMI: 24–<28 kg/m² | 517 / 1569 | 1.47 (1.29–1.66) | <0.001 |  |
| BMI: ≥28 kg/m² | 477 / 1163 | 1.41 (1.23–1.62) | <0.001 |  |
| Hypertension: No | 420 / 1824 | 1.50 (1.32–1.71) | <0.001 | 0.792 |
| Hypertension: Yes | 930 / 2140 | 1.44 (1.31–1.59) | <0.001 |  |

Values are adjusted ORs (95% CIs) per 1-SD increase in log₂-transformed SII. Interaction P values were obtained from global likelihood-ratio tests. Subgroup analyses were prespecified but considered exploratory, and no adjustment for multiple testing was applied.

# Table S5B. Temporal and reverse-causation sensitivity analyses for SII

| **Analysis** | **CHD cases / n** | **Adjusted OR per 1-SD log2(SII) (95% CI)** | **P value** |
| --- | --- | --- | --- |
| Overall cohort | 1350 / 3964 | 1.46 (1.36–1.58) | <0.001 |
| Exclude ACS at index | 1215 / 3829 | 1.45 (1.34–1.57) | <0.001 |
| Exclude newly ascertained CHD | 1107 / 3721 | 1.43 (1.32–1.56) | <0.001 |
| CHD documented ≥365 days before laboratory measurement | 899 / 3513 | 1.44 (1.32–1.57) | <0.001 |

Sensitivity analyses reduce but cannot eliminate the possibility of reverse causation inherent in the cross-sectional design.

# Table S6. Spearman correlation matrix of the nine indices and hs-CRP

| **Index** | **SII** | **PIV** | **SIRI** | **NLR** | **PLR** | **LMR** | **AIP** | **TG/HDL-C** | **Non-HDL-C** | **hs-CRP** |
| --- | --- | --- | --- | --- | --- | --- | --- | --- | --- | --- |
| SII | 1.00 | 0.84 | 0.75 | 0.80 | 0.76 | -0.54 | 0.25 | 0.25 | 0.26 | 0.68 |
| PIV | 0.84 | 1.00 | 0.85 | 0.56 | 0.59 | -0.65 | 0.24 | 0.24 | 0.26 | 0.67 |
| SIRI | 0.75 | 0.85 | 1.00 | 0.80 | 0.46 | -0.79 | 0.25 | 0.25 | 0.26 | 0.68 |
| NLR | 0.80 | 0.56 | 0.80 | 1.00 | 0.54 | -0.59 | 0.23 | 0.23 | 0.23 | 0.60 |
| PLR | 0.76 | 0.59 | 0.46 | 0.54 | 1.00 | -0.68 | 0.20 | 0.20 | 0.22 | 0.57 |
| LMR | -0.54 | -0.65 | -0.79 | -0.59 | -0.68 | 1.00 | -0.22 | -0.22 | -0.24 | -0.59 |
| AIP | 0.25 | 0.24 | 0.25 | 0.23 | 0.20 | -0.22 | 1.00 | 1.00 | 0.62 | 0.34 |
| TG/HDL-C | 0.25 | 0.24 | 0.25 | 0.23 | 0.20 | -0.22 | 1.00 | 1.00 | 0.62 | 0.34 |
| Non-HDL-C | 0.26 | 0.26 | 0.26 | 0.23 | 0.22 | -0.24 | 0.62 | 0.62 | 1.00 | 0.35 |
| hs-CRP | 0.68 | 0.67 | 0.68 | 0.60 | 0.57 | -0.59 | 0.34 | 0.34 | 0.35 | 1.00 |

AIP and TG/HDL-C are mathematically monotonic and therefore have a Spearman correlation of 1.00; they should not be interpreted as independent information sources.

# Table S7A. Direct paired comparison of SII with other markers

| **Comparator** | **Marker class** | **Corrected SII-minus-comparator ΔAUC (95% CI)** | **P value** |
| --- | --- | --- | --- |
| PIV | Inflammatory | +0.000 (−0.004 to 0.004) | 0.964 |
| SIRI | Inflammatory | −0.001 (−0.006 to 0.004) | 0.717 |
| NLR | Inflammatory | +0.003 (−0.001 to 0.007) | 0.112 |
| PLR | Inflammatory | +0.007 (0.003 to 0.011) | 0.002 |
| LMR | Inflammatory | +0.005 (−0.001 to 0.011) | 0.106 |
| AIP | Lipid-derived | +0.014 (0.008 to 0.021) | <0.001 |
| TG/HDL-C | Lipid-derived | +0.014 (0.008 to 0.021) | <0.001 |
| Non-HDL-C | Lipid-derived | +0.015 (0.008 to 0.022) | <0.001 |
| hs-CRP | Benchmark | +0.011 (0.006 to 0.016) | <0.001 |

Pairwise differences were calculated as the optimism-corrected AUC of the SII-augmented model minus that of each comparator model; positive values favor SII. Estimates were based on 500 nonparametric participant-level paired bootstrap samples, with identical resampling indices used across models. Location-shifted 95% confidence intervals were obtained by shifting the percentile limits of the apparent paired ΔAUC distribution by the estimated mean optimism. Two-sided P values were calculated using a normal approximation based on the optimism-corrected AUC difference and its paired-bootstrap standard error. All P values are nominal and were not adjusted for multiple comparisons; therefore, they should be interpreted together with the effect estimates and confidence intervals rather than as evidence of a biomarker hierarchy. AIP and TG/HDL-C produced identical estimates because AIP is a logarithmic transformation of TG/HDL-C and should not be regarded as independent evidence. AIP, atherogenic index of plasma; AUC, area under the receiver-operating-characteristic curve; CI, confidence interval; hs-CRP, high-sensitivity C-reactive protein; LMR, lymphocyte-to-monocyte ratio; NLR, neutrophil-to-lymphocyte ratio; PIV, pan-immune-inflammation value; PLR, platelet-to-lymphocyte ratio; SII, systemic immune-inflammation index; SIRI, systemic inflammation response index; TG/HDL-C, triglyceride-to-high-density lipoprotein cholesterol ratio.

# Table S7B. Internal validation and calibration metrics for the SII-augmented model

| **Validation metric** | **Value** |
| --- | --- |
| Apparent base AUC | 0.733 |
| Optimism-corrected base AUC | 0.729 |
| Apparent SII-model AUC | 0.748 |
| Optimism-corrected SII-model AUC | 0.744 |
| 10-fold CV SII-model AUC | 0.742 |
| 10-fold CV Brier score | 0.188 |
| 10-fold CV ICI | 0.017 |
| 10-fold CV calibration intercept | -0.001 |
| 10-fold CV calibration slope | 0.963 |
| Bootstrap-corrected calibration slope | 0.977 |
| Bootstrap-corrected Brier score | 0.188 |

Cross-validated calibration metrics complement discrimination and decision-curve analyses; they do not establish external transportability.

# Table S8. Non-linearity tests from adjusted spline models

| **Index** | **Reference (median)** | **P for non-linearity** |
| --- | --- | --- |
| SII | 559 | <0.001 |
| PIV | 241 | <0.001 |
| SIRI | 0.99 | <0.001 |
| NLR | 2.31 | <0.001 |
| PLR | 131 | 0.015 |
| LMR | 4.29 | <0.001 |
| AIP | 0.25 | <0.001 |
| TG/HDL-C | 1.80 | <0.001 |
| Non-HDL-C | 3.75 | <0.001 |

Natural cubic splines used three degrees of freedom and were fitted in the full analytical cohort; curves were displayed over the central 90% of each distribution.
